# Enhancing the Strength of an Optical Trap by Truncation

**DOI:** 10.1371/journal.pone.0061310

**Published:** 2013-04-08

**Authors:** Vanessa R. M. Rodrigues, Argha Mondal, Jayashree A. Dharmadhikari, Swapnesh Panigrahi, Deepak Mathur, Aditya K. Dharmadhikari

**Affiliations:** 1 Tata Institute of Fundamental Research, Mumbai, India; 2 Centre for Atomic and Molecular Physics, Manipal University, Manipal, India; LAAS-CNRS, France

## Abstract

Optical traps (tweezers) are beginning to be used with increasing efficacy in diverse studies in the biological and biomedical sciences. We report here results of a systematic study aimed at enhancing the efficiency with which dielectric (transparent) materials can be optically trapped. Specifically, we investigate how truncation of the incident laser beam affects the strength of an optical trap in the presence of a circular aperture. Apertures of various sizes have been used by us to alter the beam radius, thereby changing the effective numerical aperture and intensity profile. We observe significant enhancement of the radial and axial trap stiffness when an aperture is used to truncate the beam compared to when no aperture was used, keeping incident laser power constant. Enhancement in trap stiffness persists even when the beam intensity profile is modulated. The possibility of applying truncation to multiple traps is explored; to this end a wire mesh is utilized to produce multiple trapping that also alters the effective numerical aperture. The use of a mesh leads to reduction in trap stiffness compared to the case when no wire mesh is used. Our findings lead to a simple-to-implement and inexpensive method of significantly enhancing optical trapping efficiency under a wide range of circumstances.

## Introduction

The ability to focus a laser beam tightly, to the diffraction limit, can be effectively utilized to trap microscopic dielectric particles; this was established some decades ago in pioneering experiments conducted by Ashkin and co-workers [1]. Since then, optical traps have begun to be used to effectively probe a plethora of biophysical applications, ranging from single molecule biophysics and force spectroscopy of molecular motors to antigen detection at unprecedented attomolar concentration levels and manipulation of microtubules for applications like directed biomolecule assembly, and from Micro-Raman spectroscopy of nanoparticle-induced stress on optically-trapped stem cells to quantitative measurements of the biomechanical properties of healthy and diseased red blood cells (see [2]–[28], and references therein). Recent advances in optical trapping techniques have been cogently summarized by Bustamante and coworkers [29].

For a specified laser power, the parameters that affect the trap stiffness are incident beam size and the objective's numerical aperture (NA). On the basis of ray optics calculations, prevailing wisdom is that the objective should be overfilled in order to optimize trap stiffness [30]. In recent years, however, there have been suggestions that under-filling of the objective may actually enhance trap stiffness [31]–[33]. In the experiments hitherto reported, there has been a measure of ambiguity concerning the actual experimental conditions. Mahamdeh and coworkers [33] have introduced a parameter, namely the filling ratio α (defined as r/f.NA, where r is the laser beam radius and f is the objective's focal length), to circumvent these ambiguities. They have experimentally determined that a filling ratio of 0.95 (under-filling) when using an oil immersion objective (1.3 NA) optimizes the trap stiffness. If α is multiplied by the objective's NA, an effective NA can be assigned to the laser beam expansion; the optimal value of such an effective NA, NA_opt_, is the dimensionless parameter that becomes useful in comparing the performance of different objectives. The standard multi-element microscope objective has a rear aperture that limits the size of the incident laser beam. The effect that such an aperture has on the trap stiffness remains to be investigated even though, in general, the effects of hard apertures on Gaussian beam propagation are reasonably well established [34].

Here, we investigate and quantify how truncation of the incident laser beam in our optical trap affects its strength (trap stiffness). In our experiments beam truncation is achieved by utilizing apertures of various sizes to alter the laser beam radius, thereby changing the effective numerical aperture and intensity profile. We observe significant enhancement of the radial and axial trap stiffness when an aperture is used to truncate the beam compared to when no aperture was used, keeping incident power constant. Enhancement in trap stiffness persists even when the beam intensity profile is modulated. We also quantitatively probe the trap stiffness using apertures that have square cross section. Further, we measure the post-objective laser power as well as the profile of the focused beam. Our truncation method of enhancing trap stiffness is simple to implement and inexpensive; it may be of interest to many in the increasing community of users of optical traps in diverse areas of the biological and biomedical sciences.

## Materials and Methods

Our experimental set-up, in brief, consists of a 1 W Nd:YVO_4_ laser (Photop Suwtech, DPIR-2500) with a 2-mm beam that was expanded to 8 mm. This beam was coupled to an inverted microscope (Nikon TE 2000U). Optical trapping was achieved by focusing 1064 nm wavelength, linearly-polarized light from the laser through a 100×oil-immersed objective (numerical aperture 1.3) onto either a microscope slide material to be trapped or to a liquid flow cell. Details of our apparatus have been published in earlier reports pertaining to studies with red blood cells [8], [14], [16], [22], [26], [27], flagella dynamics [15], [17], stem cells [6], and Raman Tweezers [5], [9], [10]. In brief, the diameter of the focused laser spot (typically measured to be in the range of 1.0 µm) was kept much smaller than diameters of samples that are most likely to be encountered in biological applications of optical tweezers. Biological materials like cells typically have diameters of 6–8 µm or more.

**Figure 1:**
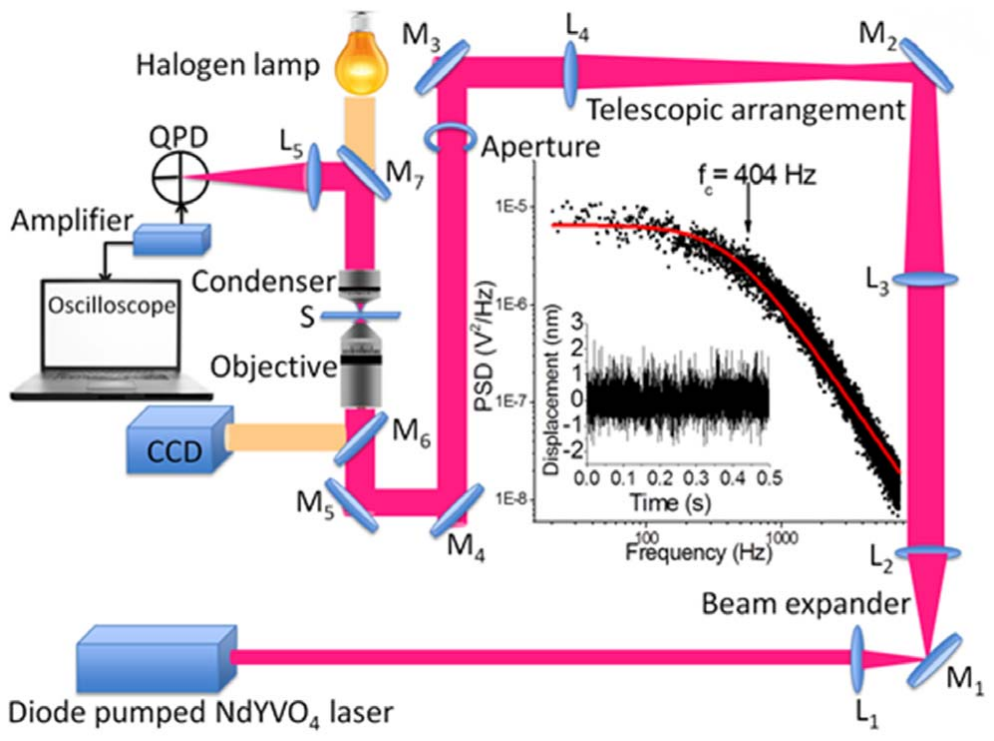
Experimental set-up of our optical trap (see text for details). The graph shows the power spectral density obtained after carrying out a Fourier transform of the displacement of a 2 µm polystyrene bead that is trapped (also shown).

The experimental set-up used in the present series of measurements that we report in the following is shown schematically in Fig.1; it comprises a 1064 nm Nd-YVO_4_ laser (Suwtech 2500) possessing a 2 mm beam diameter. The beam is expanded using lenses (L_1_ and L_2_) to 7.6 mm diameter (1/e^2^ value). This expanded beam is then passed through a 1∶1 telescopic arrangement (L_3_ and L_4_) and is routed into an inverted microscope (Nikon TE2000-U) by reflecting through a set of mirrors (M_3_, M_4_, and M_5_). The telescope permits the laser focus plane to coincide with the microscope image plane.

Optical trapping was achieved by tightly focusing the incoming laser beam by means of a 100×oil immersed objective with a NA value of 1.3. A circular aperture (made by drilling a hole in a stainless steel plate of ∼5 mm thickness) of fixed diameter (not an iris or a diaphragm) is placed in the path of the expanded beam for truncation. In order to quantitatively probe the efficacy of the thus spatially-truncated beam vis-à-vis optical trapping, we trap polystyrene beads that have been dispersed in water and placed on a #1 cover slip mounted on a sample holder (S) fixed onto a translation stage.

Trapping efficacy is quantified in terms of trap stiffness. To determine the trap stiffness we measure the power spectrum of the thermal motion of a trapped bead. For this measurement we make use of a quadrant photodiode (QPD) [2], [35]–[37] which monitors the displacement (on nanometer scales) of the trapped polystyrene bead as it undergoes Brownian fluctuations. The trapped bead is imaged (see Fig.1) using a water immersion condenser lens (Nikon, 0.9 NA) and lens L_5_ (f = 15 cm) onto a QPD (UDT SC-4D). The photodiode signals are amplified (UDT Instruments Model 431, 7 kHz bandwidth) and acquired on a 2 GHz bandwidth digital oscilloscope (Lecroy WavePro 725Zi).

To calibrate the QPD, a 2 µm polystyrene bead (Polysciences Inc.) is initially fixed to the cover slip and is subjected to a known linear displacement (along one axis) using the translation stage (Peizosystem Jena PXY 101 CAP). The corresponding QPD signal is recorded at each position; the QPD signal is plotted as a function of linear displacement and the resulting slope yields a calibration factor. In our set-up, we obtain a calibration factor of 6 mV nm^−1^.

The QPD signals allow us to measure the Brownian fluctuations of a trapped bead (in terms of displacements along two mutually perpendicular axes) as a function of time. Figure 1 shows a Fourier transform of such a displacement-time plot, which yields the power spectrum. The solid line in the spectrum is a Lorentzian fit to the experimental data. Conventionally, trapping strength is determined from such a plot in terms of the corner frequency, *f_c_*. Determination of *f_c_* is straightforward [2]: it involves drawing two lines through the data-set that is plotted. Firstly, a straight horizontal line (parallel to the frequency-axis) is drawn through the flat (linear) region of the plotted data-set and, secondly, another straight line (of slope -2) is drawn through the steeply falling part of the plotted data-set. The intersection of the two lines defines the corner frequency. Physically, this procedure represents determination of the inverse of the time for which the bead remains within the trapping volume; beyond this time, the bead diffuses out of the trapping volume. In our experiments we obtained *f_c_* by fitting to the plotted data the function [2]



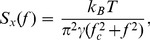
(1)where *S* denotes the power spectral density (PSD in Fig. 1), *k_B_* is Boltzmann's constant and *γ* is the drag coefficient. The trap stiffness, *k_trap_* is then readily obtained once a value of *f_c_* is deduced from the measured power spectrum:



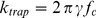
(2)where *γ* for a spherical bead is simply 6*π ηr*, where *η* denotes the viscosity of the water medium in which beads (of radius *r*) are dispersed.

It is pertinent to draw attention to a possible experimental constrain in such measurements. This constrain concerns the electronic instrumentation that is used. The bandwidth of the amplifier used in our power-spectrum measurements imposed a maximum sampling rate of 7 kHz. Consequently, the Nyquist theorem limits the frequency range that we can quantitatively determine to 3.5 kHz. However, this limiting frequency is much higher than typical values of corner frequency measured in our experiments; the power spectrum shown in Fig. 1 shows a corner frequency of 404 Hz.

The laser power at the trap was directly measured by an integrating sphere attached to a calibrated photodiode.

## Results and Discussion

The results that we present in the following were all obtained under conditions wherein polystyrene beads, of 2 µm diameter, were trapped at a height of 10 µm from the bottom of a microscope cover slip. The inset to Fig.1 shows a typical power spectral density that we obtained after Fourier transforming the bead displacement function measured by the QPD over 0.5 s intervals when there was no aperture in the laser beam path. The data shown in the inset represent the average of 10 such data sets.

**Figure 2:**
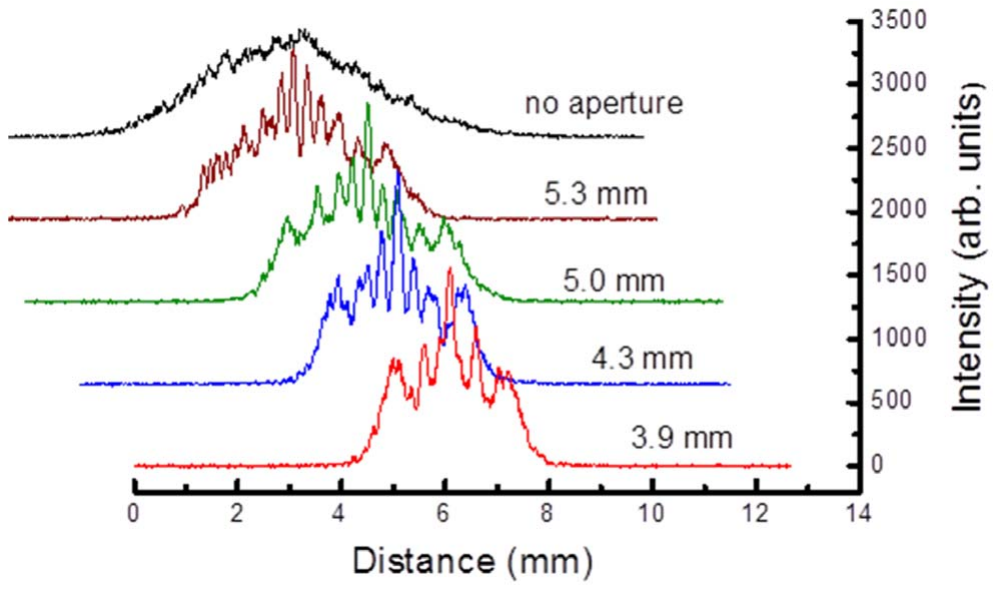
Beam profiles incident on the objective when apertures of different diameters are placed in the beam path.

We measured the beam profile of incident beam when it is truncated by various apertures using a beam profiler (Spiricon LBA-SCOR-20); typical profiles are plotted in Fig. 2. As the aperture size was reduced the beam showed modulation in the profile due to diffraction, in contrast to the smooth intensity profile that has been recently measured using a moving knife-edge [33].

In order to determine the size of the beam after 100×magnification, we introduced an aperture and replaced the QPD and lens L_5_ by a beam profiler. Initially, a 2 µm polystyrene bead was imaged to calibrate the imaging system.

**Figure 3:**
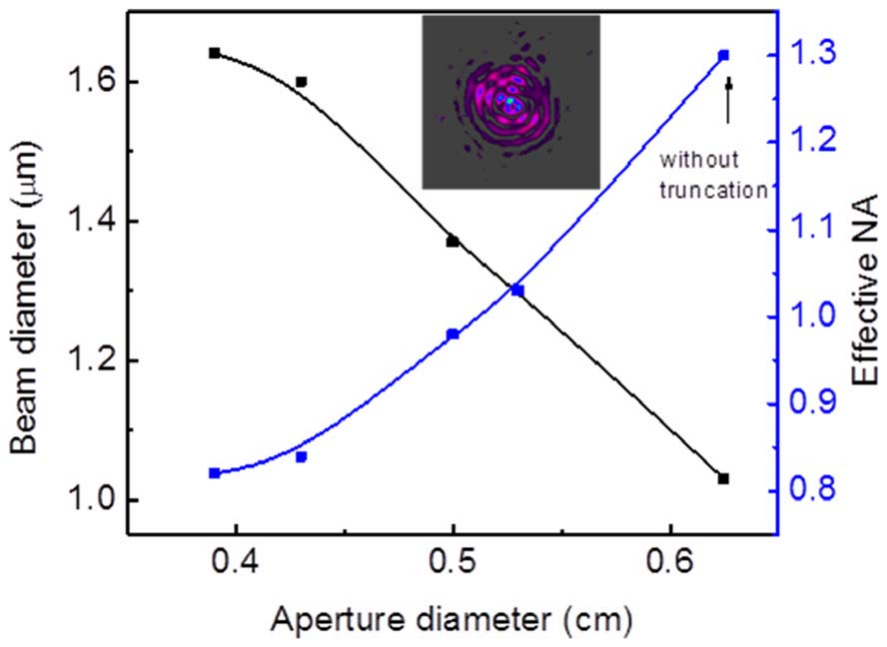
Measured focused beam diameter and corresponding effective NA as a function of aperture size. The inset shows a focused image of the spot obtained without an aperture.

The transmission through the aperture was measured to be 72.8% for a 5.3 mm aperture, 68.2% for a 5.0 mm aperture, 58.7% for a 4.3 mm aperture, and 53.2% for a 3.9 mm aperture. In each case the aperture was placed in the beam path and the size of the focus spot was measured (see Fig. 3). The inset in Fig. 3 shows an image of a focus spot of diameter 1.05 µm; this corresponds to NA of 1.3. We measured focus spot diameters and effective NA-values when different apertures were placed before the objective and typical data are presented in Fig. 3.

The measured focused beam diameter of 1.09 µm (1/e^2^ value along the *x* axis) and 1.03 (1/e^2^ value along the *y* axis) may be compared with the expected theoretical value of 1.04 µm for a Gaussian beam focused by an objective (4λ/πNA [38], where λ is the wavelength of the laser and NA is numerical aperture of the objective). The focused beam diameter without any aperture (see the image in Fig. 3) corresponds to NA of 1.3. The effective NA varied from 0.82 to 1.03 as the diameter of aperture was changed from 3.9 mm to 5.3 mm. We note that without external truncation (via an aperture) the objective's rear aperture (in our case ∼6.3 mm) introduced truncation corresponding to the datum marked with the arrow in Fig. 3.

**Figure 4:**
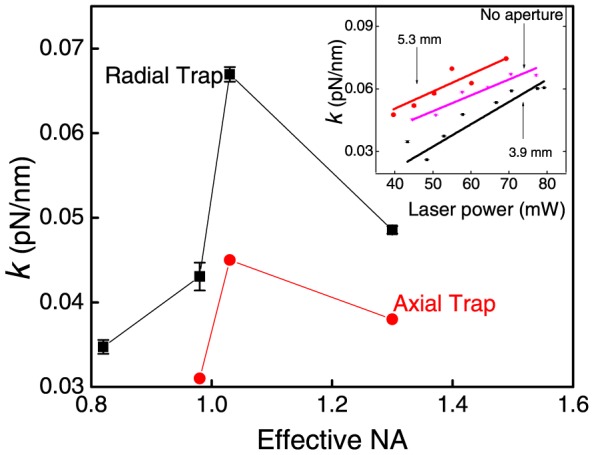
Radial and axial trap stiffness as a function of effective numerical aperture (NA) for 70 mW incident laser power. The inset shows the radial trap stiffness as a function of incident laser power for different apertures.

Values for the radial and axial trap stiffness, *k*, for 2 µm polystyrene beads were experimentally measured when apertures of different sizes were inserted in the beam path. The trap stiffness is known to depend on the bead size [33]. As noted earlier, the aperture transmission varied from 53% to 73% and, consequently, the incident power after the 100×objective was kept constant regardless of aperture size so as to obtain a meaningful comparison between truncation and no-truncation situations. We observed that when a 5.3 mm aperture was inserted (effective NA value of 1.03), the radial trap stiffness was enhanced by 40±1% compared to when there was no aperture (Fig. 4). The axial trap stiffness was also enhanced, albeit by ∼16%. This highlights our observation that enhancement of radial trap stiffness was, thus, not at the expense of axial stiffness. Similar enhancement was observed for all incident powers used in our experiments.

Our results indicate that when no aperture is in place, that is, when the objective is overfilled by 20%, the trap stiffness is distinctly lower than when a 5.3 mm aperture is introduced (under-filled condition). The power within the beam's central lobe is ∼87% for a truncation factor of 0.68 [39], whereas the power contained in the central lobe for the no-aperture case (the rear aperture of the 100×objective limited truncation to 0.8) was 91% [39]. Thus, using apertures to change the effective NA with intensity profiles as shown in Fig. 2, we obtain enhancement of trap stiffness for a 2 µm bead at NA of 1.03 (Fig. 4). Our value is ∼14% more than the recently reported result [33] where the optimal trap stiffness was obtained for an effective NA of 1.17. According to calculations based on Mie theory [33] optimal value of trap stiffness along the radial direction is obtained at NA = 1.04, which is in remarkable agreement with our measured value of NA = 1.03.

We have also carried out theoretical studies that have a bearing on the truncation-based enhancement in trap strength that has been measured by us. To this end we have utilized the computational toolbox developed by the Nieminen *et al.* [40], [41] that enables computation of the forces and torques that act on a transparent particle within an optical trap volume. The Nieminen toolbox [41] uses generalized Lorentz-Mie theory to do so and, hence, can be readily applied to particle sizes that lie between the Rayleigh regime and the ray-optics regime. A scattering approach is utilized wherein knowledge of our incident beam and the outgoing beam to compute the optically-generated forces that act on the trapped bead. The incident beam is characterized in terms of an optical field written in terms of vector spherical wavefunctions which are a general solution of the Helmholtz equation. The coefficients are deduced by least-square matching the far-field with that of the incident beam being focused by our microscope objective. The T-matrix method is used in order to characterize the scattering by the trapped bead in terms of particle properties like size, shape, refractive index and the incident light's wavelength. The coefficients of the beam emerging from our optical trap are then readily obtained using **P** = **T A**, where **A** and **P** are, respectively, the coefficient matrices of the incident and scattered beam.

The optical forces result from transfer of momentum to the trapped bead in the course of scattering. By considering momentum transfer to different parts of our spherical bead, the Nieminen toolbox [41] allows us to obtain the functional dependence of force on spatial coordinates, with the slope of this curve yielding the stiffness of our optical trap.

**Figure 5:**
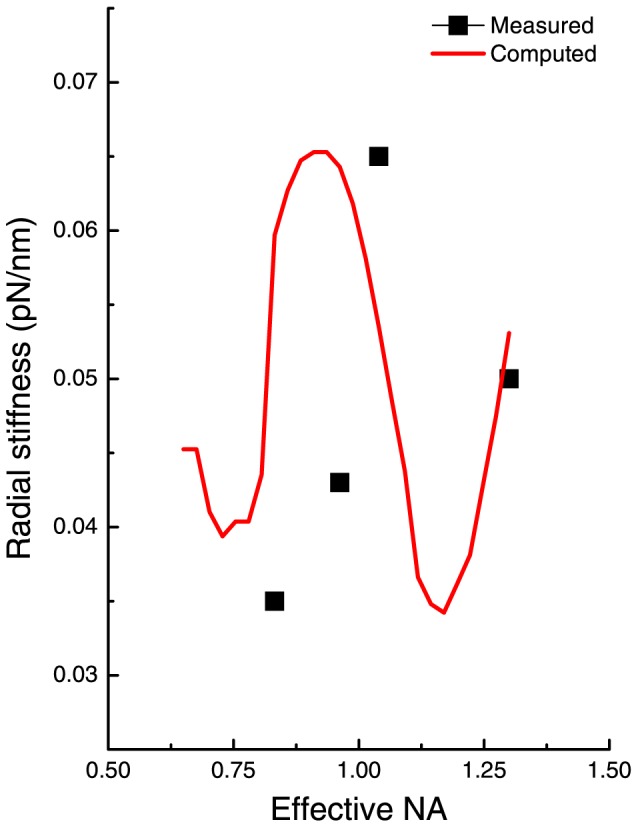
Radial trap stiffness computed using the Nieminen toolbox (see refs. [38], [39]). The line denotes the computed results while the solid squares are measured values. Our computations used the following parameters: the refractive index of our polystyrene beads was 1.57; the beads were dispersed in water whose refractive index was taken to be 1.326; the wavelength of the trapping laser was 1.064 µm.

In our computations, a Gaussian beam with y-polarization was assumed. We followed the modification to the Nieminen toolbox described in [33] to allow for an exit pupil. The Nieminen toolbox allows for hard truncation by limiting the incident beam within a cone angle. This was used in our case, where the trap stiffness was calculated for truncation angles corresponding to a range of effective NA values from 0.7 to 1.3. This closely approximated our experimental situation where truncation was achieved using a hard aperture. The change in trap stiffness with effective NA that we thus obtained is found to be in qualitative agreement with our measured data (Fig. 5). The maximum stiffness was computed to occur at an effective NA of 0.93, close to the experimental value of 1.03. We note that a similar difference between computed and measured NA-values has been observed in the only earlier study that we know of [33]; such difference may be attributed to spherical aberration at the glass-water interface which causes the effective NA to become lower.

**Figure 6:**
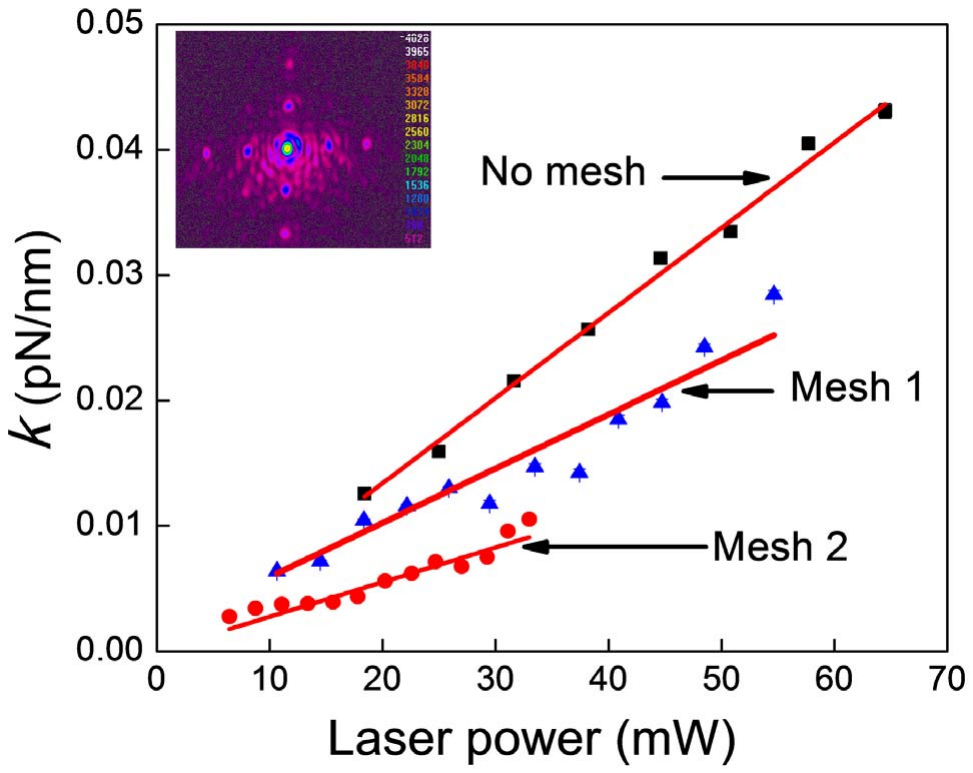
Radial trap stiffness as a function of increasing laser power for different meshes. The inset shows the beam profile of the focus spot when a mesh of size 178 µm×178 µm (Mesh 1) was inserted in the beam path. The apparent “noise” around the central spot is attributed to inevitable diffraction effects arising from the periodic apertures presented by the mesh.

Having established that enhancement of stiffness of a single trap is achieved simply by introducing an aperture (without varying laser beam size which also alters the NA value [33]), we now explore a different method of altering NA values so as to create multiple traps. We do so by introducing a wire mesh in the beam path. Our mesh was made of nickel wire of 95 µm thickness. In earlier studies we have demonstrated generation of multiple traps using a wire mesh [36] where we measured the trap stiffness (using 1 µm beads) at locations of first order diffraction spots to be typically ∼0.002 pN nm^–1^; the trap stiffness at the zero-order location was 0.0038 pN nm^–1^. In the present series of experiments, we used two different meshes to measure the trap stiffness for the zero-order diffracted spot as a function of incident laser power. The transmission of the incident laser power was 58% for Mesh 1 (a square mesh of dimensions 178 µm×178 µm) and was 35% for Mesh 2 (also a square mesh, of dimensions 65 µm×65 µm). The zero-order diffracted spot size measured for Mesh 1 (with a 100×objective) was 2.0 µm and an image is shown in the inset of Fig. 6. The effective NA was estimated to be 0.97 and 0.48 for Mesh 1 and Mesh 2, respectively.

The laser power after the objective was measured both in the presence and absence of the mesh and the trap stiffness was measured using a 2 µm bead. Plots of trap stiffness as a function of laser power after the objective, with and without the mesh (Fig. 6), indicate that the trap stiffness is smaller (by a factor of 1.26) for Mesh 1 compared to that when no mesh was used (at 18 mW incident power). For Mesh 2, the trap stiffness was still smaller (by a factor of 2.6) compared to without mesh. Thus, in the case of square aperture elements like meshes, we observe a reduction in trap stiffness compared to that when no such element is used for same transmitted power. We attribute this to a reduction in the total incident laser power in the zero-order spot. However, the trap stiffness of the zero-order spot is an order of magnitude more than that of first-order diffracted spot [36].

It is instructive to compare data presented in Figs. 4 and 6. The former data set pertains to the use of circular apertures whereas the latter is for square apertures. Each data set pertains to a distinct range of NA values, with those obtained using square apertures being for smaller values of NA. Nevertheless, it is clear that the values of trap stiffness that are obtained using the two types of apertures are mutually consistent. Smaller NA values of the type obtained with square apertures lead to trap stiffness values in the range 0.002–0.025 pN/nm (Fig. 6) while corresponding values for circular apertures are much larger, lying in the range 0.03–0.07 pN/nm (Fig. 4). The effective NA value is, clearly, the parameter of importance in quantifying the trap stiffness.

## Summary

In summary, we have observed as much as 40% enhancement in radial trap stiffness when the laser beam is truncated before the objective (under filling) using a circular aperture compared to the case when no aperture is inserted. The corresponding enhancement in axial stiffness is as much as 16%. With increase in truncation the trap stiffness reduces. The important role played by effective NA in determining trap stiffness is highlighted by our results obtained when the laser beam is truncated using circular and square apertures.

Our method is very simple to implement compared to other methods that use variable beam expanders. It is also the most inexpensive of possible methods.

From an optics perspective our results open opportunities for further work on determining the extent and magnitude of aberrations that may be introduced in our trapping apparatus that would contribute to the enhancement of stiffness that we report here. The challenge would be to overcome the difficulties associated with identifying and quantitatively determining such aberrations. We note that the use of apertures of different sizes and shapes has started to find utility in altering lateral and axial stiffness in trap, which is likely to be of critical importance in the development of holographic tweezers [42]. On a more general level, it is hoped that the work that we have presented here will stimulate efforts to gain theoretical insights into how the incident beam's intensity profile affects trap stiffness when an aperture is introduced.
